# SL-HarDNet: Skin lesion segmentation with HarDNet

**DOI:** 10.3389/fbioe.2022.1028690

**Published:** 2023-01-05

**Authors:** Ruifeng Bai, Mingwei Zhou

**Affiliations:** ^1^ Changchun Institute of Optics, Fine Mechanics and Physics, Chinese Academy of Sciences, Changchun, China; ^2^ University of Chinese Academy of Sciences, Beijing, China; ^3^ Department of Dermatology, China-Japan Union Hospital of Jilin University, Changchun, China

**Keywords:** skin lesion diagnosis, dermoscopy images, skin lesion segmentation, deep convolutional neural network, SL-HarDNet

## Abstract

Automatic segmentation of skin lesions from dermoscopy is of great significance for the early diagnosis of skin cancer. However, due to the complexity and fuzzy boundary of skin lesions, automatic segmentation of skin lesions is a challenging task. In this paper, we present a novel skin lesion segmentation network based on HarDNet (SL-HarDNet). We adopt HarDNet as the backbone, which can learn more robust feature representation. Furthermore, we introduce three powerful modules, including: cascaded fusion module (CFM), spatial channel attention module (SCAM) and feature aggregation module (FAM). Among them, CFM combines the features of different levels and effectively aggregates the semantic and location information of skin lesions. SCAM realizes the capture of key spatial information. The cross-level features are effectively fused through FAM, and the obtained high-level semantic position information features are reintegrated with the features from CFM to improve the segmentation performance of the model. We apply the challenge dataset ISIC-2016&PH2 and ISIC-2018, and extensively evaluate and compare the state-of-the-art skin lesion segmentation methods. Experiments show that our SL-HarDNet performance is always superior to other segmentation methods and achieves the latest performance.

## 1 Introduction

Skin cancer has become the highest incidence of cancer in the world (Fitzmaurice et al., 2018). In the United States, there are 5.4 million new skin cancer cases each year (Rogers et al., 2015). Melanoma is the most dangerous skin cancer (Mermelstein and Riesenberg, 1992). In 2020, there are about 100,350 new cases of melanoma in the United States, and the number of deaths is more than 6500 (Mathur et al., 2020). The 5-year survival rate of patients with advanced malignant melanoma is only 15%, while the final cure rate of patients with early stage is 95% (Barker and Postow, 2014). Therefore, the determination of melanoma lesion area and the diagnosis of benign and malignant, early and late stages play an important role in the treatment of melanoma patients.

At present, dermatologists mainly diagnose by referring to patients’ dermoscopy images. Dermatoscopy is one of the important means to improve the diagnostic accuracy and reduce the death of skin cancer (Kittler et al., 2002). During the diagnosis, the doctor visually analyzes the lesion area in the dermoscopy image. They consume a lot of time and energy in the process of repeatedly viewing dermoscopy images (Weese and Lorenz, 2016), and prone to missed diagnosis or misdiagnosis. Therefore, it is necessary to design an automatic and accurate segmentation algorithm for dermoscopy images to help dermatologists solve the above problems and improve the accuracy and efficiency of skin lesion diagnosis.

**FIGURE 1:**
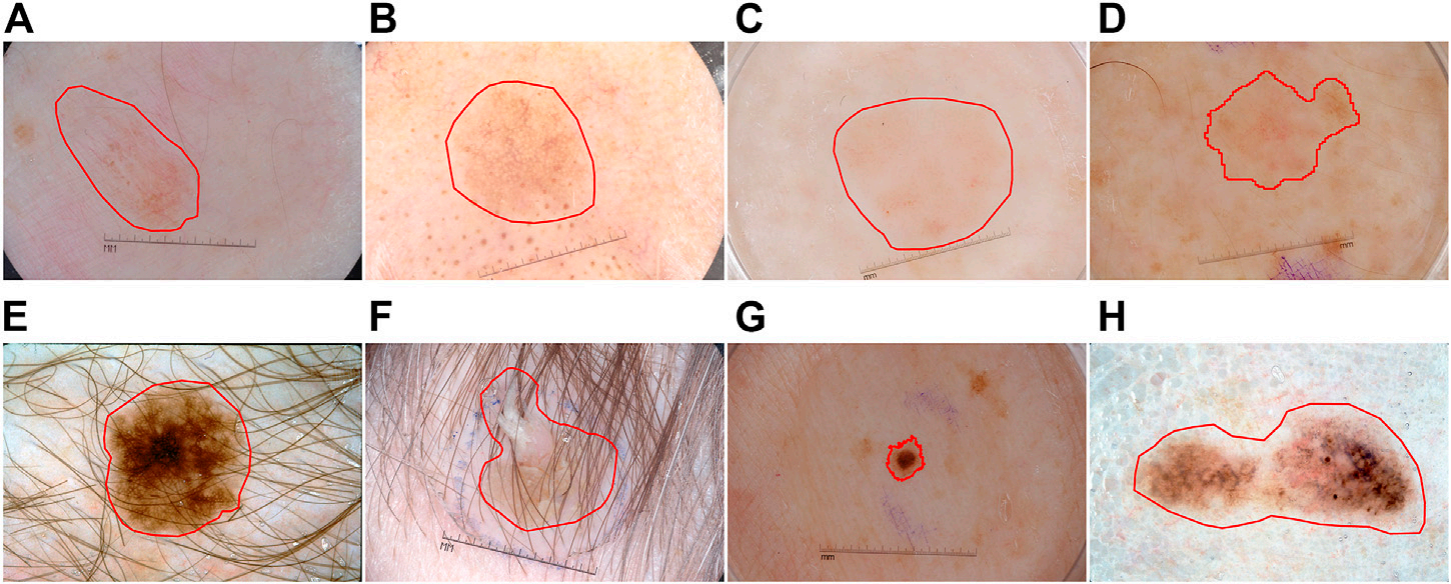
Typical skin lesion segmentation images: **(A–D)** the contrast between the lesion and the surrounding skin is low, **(E–F)** occlusion by hair and bubbles, **(G–H)** characterized by diversity.

**FIGURE 2:**
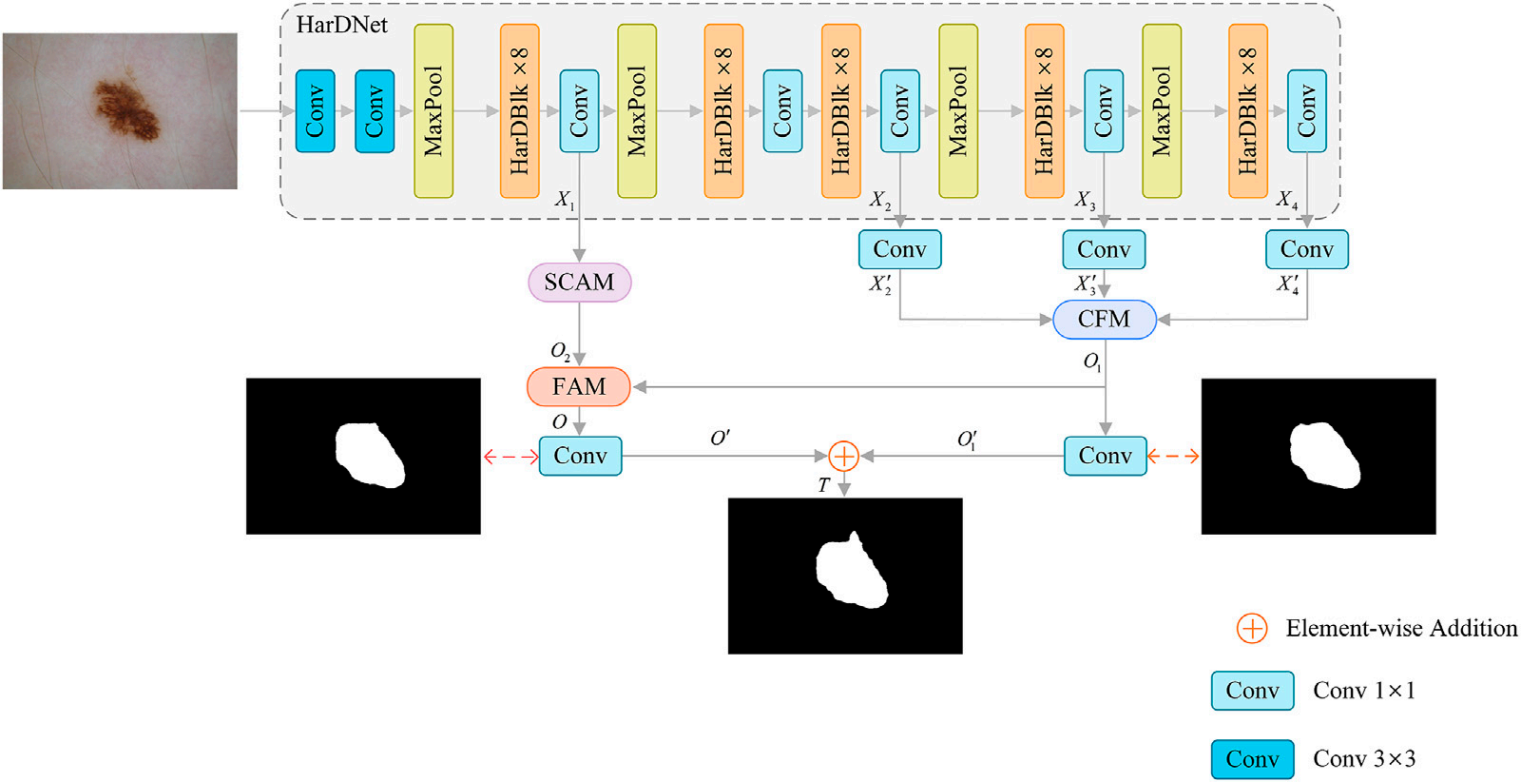
Framework of our SL-HarDNet, which consists of four parts: HarDNet backbone network, cascaded fusion module (CFM), spatial channel attention module (SCAM), feature aggregation module (FAM). HarDNet backbone is used as the encoder. CFM aggregates different scale features. SCAM obtains key location information. FAM fuses high-level and low-level features.

**FIGURE 3:**
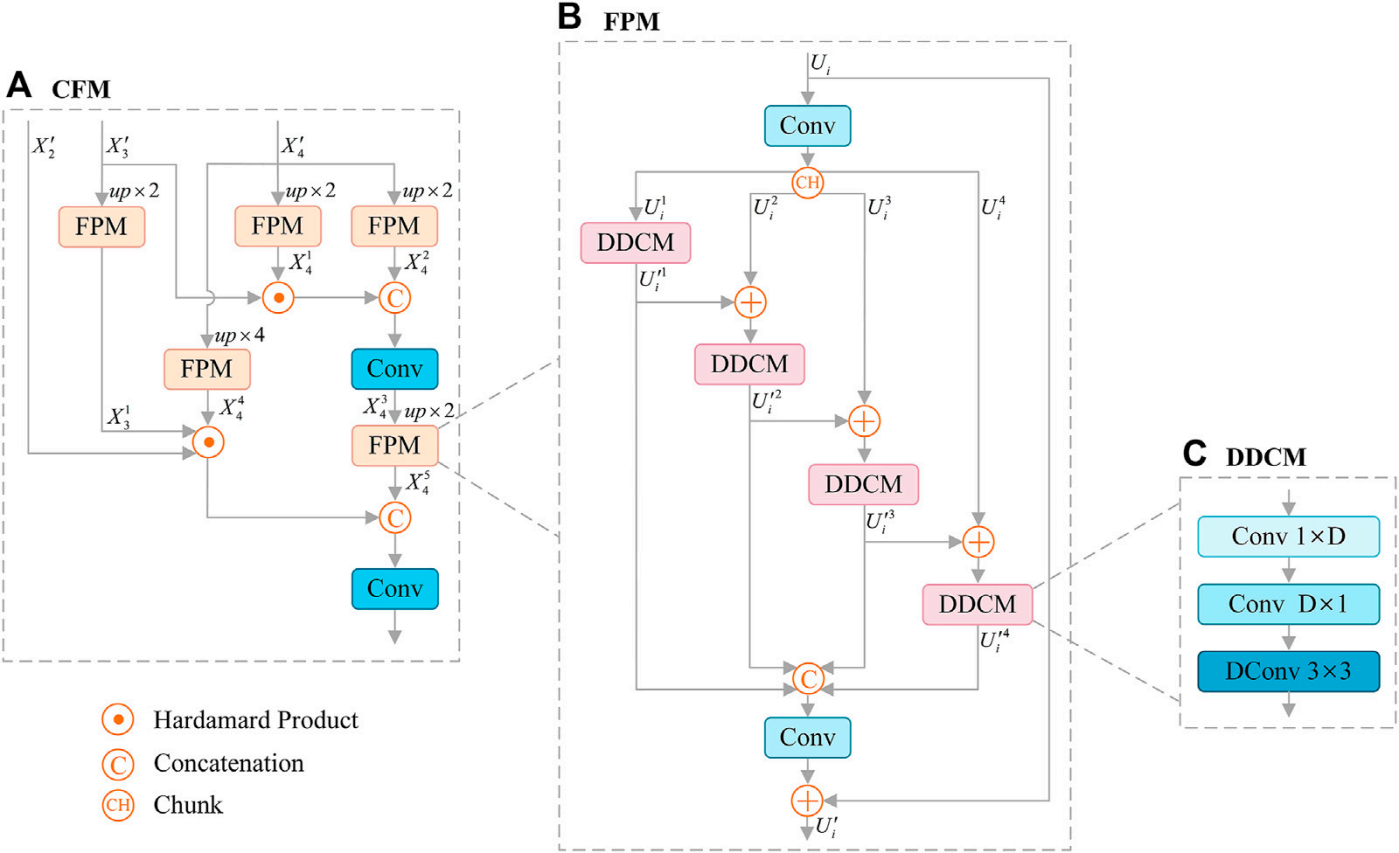
Architecture of Cascaded Fusion Module (CFM) **(A)**, which consists of Feature Pyramid Module (FPM) **(B)**, and FPM contains Depthwise Dilated Convolutions Module (DDCM) **(C)** to extract key features effectively. Dconv: Dilated Convolution.

The automatic segmentation task of dermoscopy images is used to detect the location and boundary of skin lesions. However, due to the following three reasons, segmentation is challenging: 1) The low contrast between the lesion area and the surrounding skin of the image results in blurred boundary of the lesion (see Figures 1A–D), 2) the skin lesion area gets an occlusion by hair and bubbles (see Figures 1E,F), 3) the skin lesion area is characterized by diversity, irregular shape and uneven color distribution (see Figures 1G,H).

Many efforts are devoted to overcoming these challenges. In the early stage, traditional methods based on various manual features are employed. However, the unique performance of skin lesions cannot be captured by hand-made features, resulting in poor segmentation performance of skin lesions when large changes occur. In recent years, with the continuous development of convolutional neural networks, this problem has been solved to some extent (Yu et al., 2016; Yuan et al., 2017). However, due to the lack of global context modeling, these models are insufficient to address the challenge of skin lesion segmentation. In order to solve the above problems, this paper contributes three aspects:

1) We propose a novel dermoscopy image segmentation model, termed SL-HarDNet. We adopt HarDNet as the backbone network of the model to extract more powerful and key features.
2) According to the backbone network, we design three components. Specifically, Cascaded Fusion Module (CFM) effectively extracts high-level semantic features and spatial location information of skin lesions through internal Feature Pyramid Module (FPM) and progressive methods. Meanwhile, Spatial Channel Attention Module (SCAM) enhances the extraction of channel and spatial information, which obtains the details of skin lesions and effectively reduces the error information in low-level features. Feature Aggregation Module (FAM) focuses on local information and global semantic information of the lesion area.
3) Finally, we conduct extensive experiments on ISIC-2016 & PH2 and ISIC-2018 datasets to evaluate the performance of our SL-HarDNet. Compared with the state-of-the-art model for skin lesion segmentation, our model has superior performance. This shows that our model has more prominent segmentation performance for skin lesions with different sizes, irregular, hair occlusion and blurred boundaries.

## 2 Related work

### 2.1 Traditional methods

In the early studies of dermoscopy images, the segmentation of lesions is mainly based on the classical digital image method. Usually, it can be divided into four categories: threshold method, region method, boundary method and active contour method. When the color and texture characteristics of the lesion area in the dermoscopy images are significantly different from those of the surrounding skin, the segmentation methods based on threshold can achieve good segmentation results. The commonly used threshold-based segmentation algorithms include local threshold (Ma et al., 2010), Otsu threshold (Zhou et al., 2015) and Gaussian mixture (Greggio et al., 2012). Alcón et al. (2009) proposed a threshold processing scheme for skin lesion images, which proves that Otsu threshold method could over-segment skin lesion areas. Region-based image segmentation methods can directly adopt similarity to segment the lesion area, which is simple and effective in suppressing noise interference. Among them, region growing (Javadpour and Mohammadi, 2016), region splitting and merging (Hancer et al., 2017) are the most commonly used methods. The boundary method can identify and locate the sharp discontinuous points in the image, which is conducive to the identification of image artifacts (Sakamoto et al., 2017). The common boundary methods are Prewitt filter (Chaple et al., 2015), Sobel filter (Kalra and Chhokar, 2016), Canny operator (Nikolic et al., 2016) and so on. These methods usually have the disadvantages of easy noise interference, large amount of calculation and poor local boundary segmentation. The segmentation method based on active contour is to represent the lesion boundary by continuous curve, and then defines the energy function, which transforms the image segmentation problem into the minimum problem of solving the energy functional. Although this method obtains continuous boundary contour, the calculation process is complex, time consuming and sensitive to noise (Xie and Bovik, 2013). In summary, the robustness of the early skin lesion segmentation methods based on digital image processing needs to be improved, and it is difficult to adapt to highly variable samples in practical applications. In particular, it is unable to effectively deal with the problem of irregular lesions and low contrast in dermoscopy images. Early segmentation algorithms are difficult to achieve satisfactory segmentation results.

### 2.2 Deep learning methods

In recent years, since Hinton and Salakhutdinov (2006) proposed the concept of deep learning, deep learning based on convolutional neural network (CNN) has achieved great success in image segmentation, image classification and target detection. Similarly, CNN has changed the development of dermoscopy image segmentation and recognition, realizing end-to-end training and prediction. The specificity, sensitivity and accuracy of skin lesion segmentation and classification are higher than those of medical professionals.

Long et al. (2015) proposed the full convolution neural network (FCN), which replaces the full connection layer with the convolution layer, so that the improved network has the ability of pixel-by-pixel prediction and solved the problem of semantic segmentation. Ronneberger et al. (2015) proposed a U-Net framework for the segmentation of medical images with small samples, which is outstanding in the medical image segmentation tasks and has become the mainstream medical image segmentation algorithm. U-Net and U-Net improved are also widely applied in skin lesion segmentation. TernausNet (Iglovikov and Shvets, 2018) uses pre-trained VGG (Szegedy et al., 2015) as coding block to improve the U-Net, which improves the accuracy of segmentation, but this method ignores the segmentation efficiency. To solve this problem, Chaurasia and Culurciello (2017) proposed the LinkNet, which adopts the residual block as the encoder, greatly reducing the number of parameters and improving the segmentation efficiency. Alom et al. (2018) presented a recurrent residual convolution neural network, which is based on recurrent residual layer of cyclic convolution to accumulate features and obtain good segmentation results. The above methods significantly enhance the extraction of skin lesion features by the segmentation network, but cannot obtain sufficient global information to achieve higher segmentation accuracy and cannot deal with fuzzy boundaries. Koohbanani et al. (2018) realized the overall prediction of skin lesions by combining multi-scale convolution with multiple different depth models. However, due to the integration of multiple models, resulting in a sharp increase in the number of parameters, network convergence time is greatly extended.

In the process of skin lesion segmentation, accurate feature extraction is the key to achieve high-precision segmentation. A large number of studies focus on the design of feature extractor. Among them, Al-Masni et al. (2018) proposed a full-resolution segmentation model for the irregular and ambiguous boundary of skin lesions, which improves the segmentation accuracy. Xie F. et al. (2020) proposed a convolution neural network segmentation method based on attention mechanism, which obtains the detailed features of lesions by fusing multi-branch outputs, but the fuzzy boundary of skin lesions is still difficult to identify. In addition to using standard convolution and depth separable convolution, deconvolution is also used in skin lesion segmentation tasks. Yuan and Lo (2017) introduced deconvolution method in the color space of dermoscopy image, and achieved certain results in lesion segmentation. However, it should be noted that deconvolution operations require high computational costs, greatly increasing the consumption of computing resources, and still fail to effectively address the problem of fuzzy boundaries (Fan et al., 2019). Vision transformer is also widely used in the segmentation of skin lesions (Dosovitskiy et al., 2020), and has achieved good segmentation results (Wang et al., 2021; Cao et al., 2022). However, these methods do not effectively consider the boundary and global information of skin lesions, resulting in insufficient segmentation performance in extreme cases.

In summary, the segmentation methods of dermoscopy image based on deep neural network have remarkable effects, but there are still many challenges in deep modeling. Most of the existing dermoscopy segmentation models have insufficient feature information extraction and less edge information retained in the segmentation results. Therefore, for the segmentation of dermoscopy images, we design a new lesion segmentation framework based on HarDNet (Chao et al., 2019), which can accurately locate the boundary of skin lesions even in extreme cases.

## 3 Methods

### 3.1 Overall architecture

The proposed SL-HarDNet is mainly composed of four parts: HarDNet backbone network, cascaded fusion module (CFM), spatial channel attention module (SCAM), feature aggregation module (FAM). Figure 2 summarizes the overall structure of the SL-HarDNet. Specifically, HarDNet is improved by DenseNet (Huang et al., 2017) dense block, which has efficient reasoning speed and high precision segmentation performance. CFM aggregates different scale features by progressive method, and effectively obtains semantic information from skin lesions in HarDNet. SCAM enhances spatial and channel information extraction and modeling. FAM efficiently fuses semantic information from CFM and spatial information from SCAM.

The input image size is $I\in R^{H\times W\times 3}$ . Firstly, four different scale features ${\left\{X_{l}\right\}}_{l=1}^{4}$ are extracted from HarDNet backbone network. Here, $X_{1}$ represents the lowest feature and $X_{4}$ represents the deepest feature ( $X_{l}\in R^{\frac{H}{2^{l+1}}\times \frac{W}{2^{l+1}}\times C_{l}}$ ; $C_{l}\in \left\{128,320,640,1024\right\}$ ). Then, $X_{2}$ , $X_{3}$ and $X_{4}$ are processed by 1×1 convolution, and the number of channels is reduced to 32. The processed feature ( $X_{2}^{\prime }$ , $X_{3}^{\prime }$ and $X_{4}^{\prime }$ ) is input into CFM to complete the effective fusion of multi-scale features, and the feature map $O_{1}\in R^{\frac{H}{8}\times \frac{W}{8}\times 32}$ is generated. Meanwhile, after the lowest feature $X_{1}$ is processed by SCAM, the feature map $O_{2}\in R^{\frac{H}{4}\times \frac{W}{4}\times 128}$ is obtained. Then, $O_{1}$ and $O_{2}$ are aligned and input into FAM for feature aggregation to obtain feature $O\in R^{\frac{H}{8}\times \frac{W}{8}\times 32}$ containing semantic location information. Then, O and $O_{1}$ are processed by 1×1 convolution layer, and the processed features are $O^{\prime }$ and $O_{1}^{\prime }$ respectively. Finally, the obtained $O^{\prime }$ and $O_{1}^{\prime }$ are further fused and the 1×1 convolution layer is processed to obtain the feature T as the final segmentation feature map. During training, the loss function mainly consists of two parts, one is the loss between the segmentation result $O^{\prime }$ and Ground Truth to optimize the segmentation result of skin lesions. The other part is the loss between the result $O_{1}^{\prime }$ generated by CFM and Ground Truth, which is used to supervise CFM.

### 3.2 HarDNet encoder

Dermoscopy images are inevitably disturbed by hair, bubbles, blood vessels and light. At present, the segmentation of skin lesions usually adopts the strategy of deep supervision. The multi-scale feature information (Abraham and Khan, 2019) and cascade architecture (Xie Y. et al., 2020; Jin et al., 2021) are introduced into the network to achieve more precise segmentation results. HarDNet is improved by DenseNet, and the dense connection of the DenseNet leads to a large amount of memory occupation and an increase in computation. HarDNet reduces the connection between most layers on the basis of DenseNet to improve the inference speed, and increases the channel width of the key layer to compensate for the loss of accuracy. Furthermore, HarDNet has the advantages of feature reuse and deep supervision, so this paper chooses HarDNet as the backbone of feature extraction. Precisely, HarDNet68 (Chao et al., 2019) is used as the backbone network. HarDNet68 is mainly composed of five 8-layer harmonic dense blocks (HarDBlk). Among them, the output feature sizes of the second and third HarDBlk × 8 are the same, and the fourth and fifth HarDBlk × 8 output feature map scale is halved in turn. There is a 1×1 convolution behind each HarDBlk × 8 to adjust the number of channels. In order to make full use of the information at different scales, this paper extracts multi-scale features from the first, third, fourth and fifth HarDBlk × 8 of HarDNet68, and the corresponding features ( $i.e.X_{1}$ , $X_{2}$ , $X_{3}$ and $X_{4}$ ) are obtained by 1×1 convolution.

### 3.3 Cascaded Fusion Module

Skin lesions usually show irregular shapes. In order to better extract the semantic feature information, we adopt the effective feature extraction structure Cascaded Fusion Module (CFM), as shown in Figure 3A. In particular, we introduce Feature Pyramid Module (FPM) to encode multi-scale features (see Figure 3B), which combines dilated convolution and reverse bottleneck convolution to extract key features more effectively. FPM is a module based on dilated reverse bottleneck convolution, which is composed of 1×1 convolution layer and Depthwise Dilated Convolutions Module (DDCM) (see Figure 3C), and applies residual connection.

Here, $X_{2}^{\prime }$ , $X_{3}^{\prime }$ and $X_{4}^{\prime }$ are input into CFM respectively. $X_{1}$ contains rich color, shape and other feature information, lack of semantic information, provides detailed spatial information. $X_{2}^{\prime }$ , $X_{3}^{\prime }$ and $X_{4}^{\prime }$ include abundant semantic information and provide advanced features. We define FPM as $FPM\left(\cdot \right)$ , DDCM denotes $DDCM\left(\cdot \right)$ , $F_{3\times 3}\left(\cdot \right)$ is 3×3 convolution layer with padding set to 1, and adopt batch normalization (Ioffe and Szegedy, 2015) and ReLU (Glorot et al., 2011). CFM is mainly composed of two cascaded parts, as follows:

1) In the first cascade, the deepest feature $X_{4}^{\prime }$ is up-sampled 2 times to the same size as $X_{3}^{\prime }$ , and then the results are introduced into the corresponding $FPM\left(\cdot \right)$ to obtain $X_{4}^{1}$ and $X_{4}^{2}$ , respectively. Then $X_{4}^{1}$ and $X_{3}^{\prime }$ are multiplied, and the obtained results are concatenate with $X_{4}^{2}$ . Finally, the fused feature map $X_{4}^{3}$ is obtained by the 3×3 convolution layer $F_{3\times 3}\left(\cdot \right)$ . The process can be summarized as follows:

$$X_{4}^{3}=F_{3\times 3}(Concat(FPM(X_{4}^{\prime })⊙X_{3}^{\prime },FPM(X_{4}^{\prime })))$$ (1)

Where “ ⊙ ” represents Hadamard product, and $Concat\left(\cdot \right)$ represents concatenate operation along the channel dimension.

2) The treatment in the second part is similar to that in the first part. First, we upsample ${X_{3}}^{\prime }$ and $X_{4}^{3}$ 2 times, and ${X_{4}}^{\prime }$ 4 times upsample so that they have the same size as ${X_{2}}^{\prime }$ . Then, the key features $X_{3}^{1}$ and $X_{4}^{4}$ are extracted by using the corresponding $FPM\left(\cdot \right)$ , and $X_{3}^{1}$ , $X_{4}^{4}$ and ${X_{2}}^{\prime }$ are multiplied to concatenate the obtained mapping with the $X_{4}^{5}$ obtained by the corresponding $FPM\left(\cdot \right)$ processing. Finally, we introduce the concatenate feature mapping into the 3×3 convolution layer ( $i.e.F_{3\times 3}\left(\cdot \right)$ ) to reduce the dimension, and finally get $O_{1}\in R^{\frac{H}{8}\times \frac{W}{8}\times 32}$ , which is the output of CFM. The process is as follows:

$$O_{1}=F_{3\times 3}(Concat(FPM(X_{3}^{\prime })⊙FPM(X_{4}^{\prime })⊙X_{2}^{\prime },FPM(X_{4}^{3})))$$ (2)

It should be noted that the feature ( $U_{i}\in \left\{{X_{2}}^{\prime },{X_{3}}^{\prime },{X_{4}}^{\prime }\right\}$ ; $i\in \left\{1,2,3\right\}$ ) is treated more deeply in FPM. Firstly, the input ( $U_{i}\in \left\{{X_{2}}^{\prime },{X_{3}}^{\prime },{X_{4}}^{\prime }\right\}$ ; $i\in \left\{1,2,3\right\}$ ) of FPM is processed by 1×1 convolution layer $F_{1\times 1}\left(\cdot \right)$ , and then the obtained results are equally divided into four chunk features ( $U_{i}^{1}$ , $U_{i}^{2}$ , $U_{i}^{3}$ and $U_{i}^{4}$ ) along the channel. Then, the first chunk feature $U_{i}^{1}$ is processed by $DDCM\left(\cdot \right)$ to obtain feature ${U^{\prime }}_{i}^{1}$ . The second, third and fourth chunk features ( ( $U_{i}^{2}$ , $U_{i}^{3}$ and $U_{i}^{4}$ )) are combined with the previous chunk processed features ( $i.e.{U^{\prime }}_{i}^{1}$ , ${U^{\prime }}_{i}^{2}$ and ${U^{\prime }}_{i}^{3}$ ), and then introduced into the corresponding $DDCM\left(\cdot \right)$ to obtain ${U^{\prime }}_{i}^{2}$ , ${U^{\prime }}_{i}^{3}$ and ${U^{\prime }}_{i}^{4}$ . Then, the feature ${U^{\prime }}_{i}^{k}$ ( $k\in \left\{1,2,3,4\right\}$ ) is concatenated, and the obtained result uses the 1×1 convolution layer $F_{1\times 1}\left(\cdot \right)$ to restore the number of channels. Finally, the obtained results are combined with the initial input of FPM to obtain the final output ${U_{i}}^{\prime }$ . The operation procedure of FPM is as follows:

$$U_{i}^{k}=Chunk\left(F_{1\times 1}\left(U_{i}\right)\right)\begin{array}{cc} , & k\in \left\{1,2,3,4\right\} \end{array}$$ (3)

$${U^{\prime }}_{i}^{1}=DDCM\left(U_{i}^{1}\right)$$ (4)

$${U^{\prime }}_{i}^{j}=DDCM\left({U^{\prime }}_{i}^{\left(j-1\right)}+U_{i}^{j}\right)\begin{array}{cc} , & j\in \left\{2,3,4\right\} \end{array}$$ (5)

$${U_{i}}^{\prime }=Concat\left(F_{1\times 1}\left({U^{\prime }}_{i}^{k}\right)\right)+U_{i}$$ (6)

DDCM increases the receptive field and reduces the computational complexity without reducing the resolution of the feature map. The operation $DDCM\left(\cdot \right)$ of DDCM can be expressed as:

$$DDCM\left(x\right)=F_{3\times 3}^{D}\left(F_{D\times 1}\left(F_{1\times D}\left(x\right)\right)\right)$$ (7)

Where $Chunk\left(\cdot \right)$ denotes the splitting operation along the channel dimension. $x\in \left\{U_{i}^{1},{U^{\prime }}_{i}^{2},{U^{\prime }}_{i}^{3},{U^{\prime }}_{i}^{4}\right\}$ . $F_{1\times D}\left(\cdot \right)$ is defined as 1×D convolution layer with padding set to $\left(0,d\right)$ ( $d\in \left\{0,1,2,3\right\}$ ). $F_{D\times 1}\left(\cdot \right)$ represents D×1 convolution layer with padding set to $\left(d,0\right)$ . Similarly, $F_{3\times 3}^{D}\left(\cdot \right)$ is 3×3 dilated convolution layer with padding set to $\left(D,D\right)$ ( $D\in \left\{1,3,5,7\right\}$ ). D represents the expansion rate in $F_{3\times 3}^{D}\left(\cdot \right)$ . As shown in Figure 3B, the expansion rates in DDCM corresponding to branch ${U^{\prime }}_{i}^{k}$ ( $k\in \left\{1,2,3,4\right\}$ ) are 1, 3, 5, 7 respectively.

### 3.4 Spatial channel attention module

**FIGURE 4:**
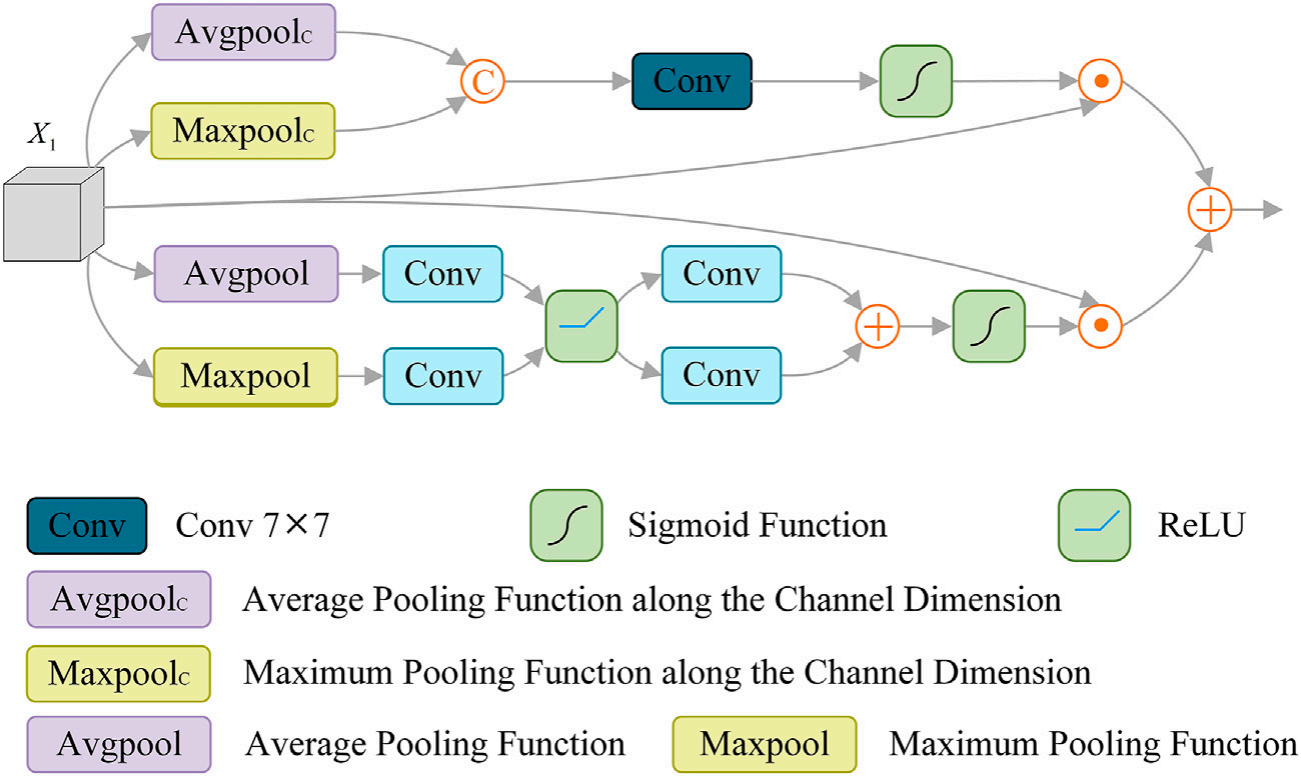
Architecture of spatial channel Attention module (SCAM).

Some methods (Zhao et al., 2017; Chen et al., 2018; Zhang et al., 2018) usually focus on high-level semantic information, while ignoring the underlying spatial details. Other methods (Jégou et al., 2017; Lin et al., 2017; Peng et al., 2017) adopt complex modules to aggregate different features, and use low-level features to refine the boundary, which solves the problem of coarse segmentation to some extent. Considering the channel attention mechanism has the advantages of increasing the correlation between different channels and improving the weight of the segmented target. At the same time, the spatial attention mechanism can correlate key features in different spaces. Therefore, we introduce the Spatial Channel Attention Module (SCAM), which is applied to enhance the extraction of channel and spatial information and effectively identify the details of skin lesions, as shown in Figure 4.

Specifically, SCAM is composed of spatial attention operation $SAM\left(\cdot \right)$ and channel attention operation $CAM\left(\cdot \right)$ , which can be expressed as:

$$O_{2}=SAM\left(X_{1}\right)+CAM\left(X_{1}\right)$$ (8)

Spatial attention operation $SAM\left(\cdot \right)$ can be written as follow:

$$SAM(x)=\sigma (F_{7\times 7}\left(Concat\right.(P_{Cavg}(x),P_{Cmax}(x))))⊙x$$ (9)

Where x represents the input, and σ is the Sigmoid function. $F_{7\times 7}\left(\cdot \right)$ denotes the 7×7 convolution layer. $P_{Cavg}\left(\cdot \right)$ and $P_{Cmax}\left(\cdot \right)$ represent average pooling function and the maximum pooling function along the channel, respectively.

Channel attention operation $CAM\left(\cdot \right)$ can be formulated as:

$$CAM\left(x\right)=\sigma (H_{1}(P_{avg}\left(x\right))+H_{2}\left(P_{\max}\left(x\right)\right))⊙x$$ (10)

Where $P_{avg}\left(\cdot \right)$ and $P_{\max}\left(\cdot \right)$ denote adaptive average pooling function and adaptive maximum pooling function, respectively. $H_{i}\left(\cdot \right)$ contains a 1×1 convolution layer that reduces the channel dimension by 16 times, and then there is a ReLU layer and another 1×1 convolution layer, so that the feature is restored to the original number of channels.

### 3.5 Feature aggregation module

**FIGURE 5:**
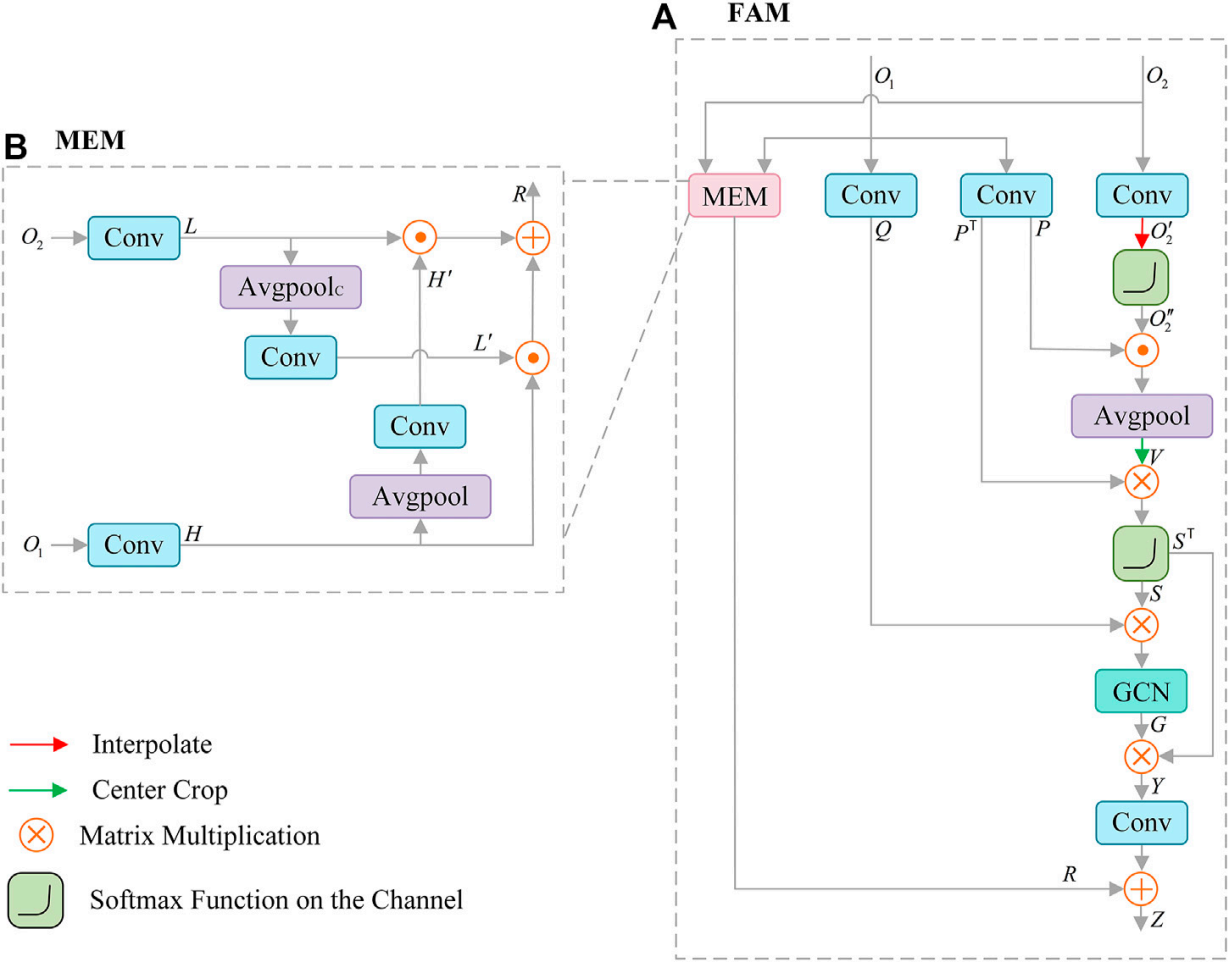
Architecture of Feature Aggregation Module (FAM) **(A)**, which consists of mutual embedding module (MEM) **(B)** to enhance the fusion of features, graph convolution (GCN) for reconstructing features.

Features from SCAM contain rich details, and the features of CFM output include high-level semantic information. To make full use of the correlation information between them, we propose Feature Aggregation Module (FAM), as shown in Figure 5A. FAM is mainly composed of graph convolution (GCN) (Lu et al., 2019), non-local operation (Wang et al., 2018; Te et al., 2020) and mutual embedding module (MEM) (Liu and Yin, 2019). FAM effectively introduces the global information through non-local operation, and adopts the key features extracted by graph convolution to construct the feature relationship, which supplements the structural information of the lesion for the extracted features. In MEM (see Figure 5B), low-level features are embedded in context information, and high-level features are embedded in spatial details, which effectively enhances the fusion of features.

The high-level semantic feature $O_{1}\in R^{\frac{H}{8}\times \frac{W}{8}\times 32}$ and the rich spatial detail feature $O_{2}\in R^{\frac{H}{4}\times \frac{W}{4}\times 128}$ are imported into FAM. First, the corresponding 1×1 convolution layer ( $i.e.F_{1\times 1}\left(\cdot \right)$ ) is used to reduce the dimension, and the resulting features are $Q\in R^{\frac{H}{8}\times \frac{W}{8}\times 16}$ and $P\in R^{\frac{H}{8}\times \frac{W}{8}\times 16}$ respectively. The process can be formulated as:

$$Q=F_{1\times 1}\left(O_{1}\right),\;P=F_{1\times 1}\left(O_{1}\right)$$ (11)

For spatial detail $O_{2}$ , the number of channels is reduced to 32 by 1×1 convolution layer ( $i.e.F_{1\times 1}\left(\cdot \right)$ ), and the feature $O_{2}^{\prime }\in R^{\frac{H}{4}\times \frac{W}{4}\times 32}$ is obtained. Then, bilinear interpolation is performed to ensure the same size as $O_{1}$ , and the Softmax function is adopted along the channel. The second channel is selected as the attention map, and the obtained feature is $O_{2}^{\prime \prime }\in R^{\frac{H}{8}\times \frac{W}{8}\times 1}$ . Next, we multiply $O_{2}^{\prime \prime }$ and P . The feature map $V\in R^{16\times 16\times 1}$ is obtained by using adaptive average pooling and center clipping. In summary, the process can be expressed as:

$$V=CP\left(P⊙\left(\delta \left(F_{1\times 1}\left(O_{2}\right)\right)\right)\right)$$ (12)

Where the $CP\left(\cdot \right)$ is adaptive average pooling and clipping operation. δ is the Softmax function.

We adopt the inner product to associate each element in V and P , the operation is as follows:

$$S=\delta \left(V⊗P^{T}\right)$$ (13)

Where “ ⊗ ” denotes the inner product operation. $P^{T}$ is the transposition of P . S represents the correlation attentional map.

The obtained S and Q are inner products, and the results are passed into the GCN (Lu et al., 2019) to get $G\in R^{16\times 16\times 1}$ . We define GCN as $GCN\left(\cdot \right)$ . Then, the inner product between G and $S^{T}$ is calculated. In this way, the graph domain feature is reconstructed into the original structural feature, and the operation process can be constructed as follows:

$$Y=S^{T}⊗GCN\left(S⊗Q\right)$$ (14)

Meanwhile, the MEM module is used to enhance the integration of $O_{1}$ and $O_{2}$ . The MEM operation can be divided into three parts:

1) The feature $O_{2}$ containing spatial information is imported into the 1×1 convolution layer, and the result $L\in R^{\frac{H}{8}\times \frac{W}{8}\times 32}$ is obtained. Then, feature L uses the average pooling along the channel and 1×1 convolution layer to obtain $L^{\prime }$ . This process can be summarized as follows:

$$L^{\prime }=F_{1\times 1}\left(P_{Cavg}\left(F_{1\times 1}\left(O_{2}\right)\right)\right)$$ (15)

2) The high-level semantic feature $O_{1}$ is operated by 1×1 convolution layer to get $H\in R^{\frac{H}{8}\times \frac{W}{8}\times 32}$ . Then, through the processing of adaptive average pooling and 1×1 convolution, the result is $H^{\prime }$ . The process can be described as:

$$H^{\prime }=F_{1\times 1}\left(P_{avg}\left(F_{1\times 1}\left(O_{1}\right)\right)\right)$$ (16)

3) The result of multiplying $H^{\prime }$ and L is fused with the result of multiplying $L^{\prime }$ and H , as follows:

$$R=\left(H^{\prime }⊙L\right)+\left(H⊙L^{\prime }\right)$$ (17)

Finally, the 1×1 convolution kernel is used to adjust the feature map Y to the same size as $O_{1}$ , and combined with the feature $R\in R^{\frac{H}{8}\times \frac{W}{8}\times 32}$ from the MEM, the output is $Z\in R^{\frac{H}{8}\times \frac{W}{8}\times 32}$ . Summary as follows:

$$Z=R+F_{1\times 1}\left(Y\right)$$ (18)

### 3.6 Loss function

In order to achieve fine segmentation, we combine Weighted Intersection Over Union loss (Loshchilov and Hutter, 2017) and Weighted Binary Cross Entropy loss (Loshchilov and Hutter, 2017) to focus on the segmentation of uncertain lesion boundaries and improve segmentation performance. Combination loss is defined as:

L=IL+BL (19)

Where IL denotes Weighted Intersection Over Union loss and BL represents Weighted Binary Cross Entropy loss. L is combination loss. Unlike the standard Intersection Over Union (IOU) loss, IL focuses on the importance of each pixel and pays more attention to hard pixel. Compared with the standard Binary Cross Entropy (BCE) loss, BL assigns higher weights to hard pixels.

The final loss consists mainly of the loss between $O_{1}$ and Ground Truth G , and the loss between $O_{2}$ and Ground Truth G . The loss between result $O_{1}$ and Ground Truth G can be expressed as:

$$L_{1}=IL\left(O_{1},G\right)+BL\left(O_{1},G\right)$$ (20)

The loss between result $O_{2}$ and Ground Truth G can be written as:

$$L_{2}=IL\left(O_{2},G\right)+BL\left(O_{2},G\right)$$ (21)

The final loss is as follows:

$$L=L_{1}+L_{2}$$ (22)

## 4 Experiments

### 4.1 Datasets

We adopt ISIC-2018 (Codella et al., 2019) and ISIC-2016 (Gutman et al., 2016) & PH2 (Mendonça et al., 2013) dermoscopy datasets to evaluate our model.

1) ISIC-2018 Dataset: The 2018 International Skin Imaging Collaboration (ISIC) skin lesion segmentation challenge dataset contains 2594 images and corresponding labels. Image resolution changes between 720×540 and 6708×4439 . Since the public test set has not yet been published, this paper applies 5-fold cross validation for fair comparison.
2) ISIC-2016&PH2 Dataset: The samples from two different centers are included to evaluate the accuracy and generalization ability of skin lesion segmentation. The ISIC-2016 contains 900 training samples and 379 validation samples, and PH2 dataset contains 200 samples. In this paper, we adopt the ISIC-2016 dataset for model training and validation, and PH2 dataset for model testing.

### 4.2 Implementation details

This study is implemented on the Pytorch and applies two Nvidia Geforce 3090 cards to complete model training. Taking into account the difference in the size of the dermoscopy image and the improvement of computational efficiency, the image is adjusted to 512×512 . Meanwhile, in order to expand the training dataset and increase the diversity of data, we carry out data enhancement, including vertical flip, horizontal flip, random rotation and gauss noise. During the training, the mini-batch size is set to 8, the initial learning rate is 1e−4 , learning rate decay policy is CosineAnnealingLR (Loshchilov and Hutter, 2016), and the optimizer adopts Adam W (Loshchilov and Hutter, 2017). More details about parameter setting in training are shown in Table 1. We train the model for 200 epochs and save the optimal segmentation performance during validation as model parameters.

**TABLE 1:** Parameter setting during the training stage.

Vertical flip	Horizontal flip	Random rotation	Gauss noise
0.5			

Input size	Epochs	Optimizer	Learning Rate(lr)	Learning rate policy
512×512	200	Adam W	1e−4	CosineAnnealingLR

### 4.3 Evaluation metrics

We adopt five performance indicators to evaluate the obtained segmentation results, including the Jaccard index (JAC), Dice coefficient (DIC), accuracy (ACC), sensitivity (SEN) and specificity (SPE). JAC is used to measure the similarity between data samples, which is proportional to the segmentation accuracy. The larger the JAC value, the higher the segmentation accuracy. DIC is usually used to evaluate the segmentation accuracy of the network. The higher the DIC value, the smaller the difference between the data, and the more accurate the segmentation. ACC, SEN and SPE are common statistical measures for evaluating binary classification performance.

### 4.4 Comparison with state-of-the-arts

#### 4.4.1 Qualitative analysis

Our models are compared with several popular skin lesion segmentation models. On the ISIC-2016&PH2 dataset, competition methods include FCN (Long et al., 2015), UNet++ (Zhou et al., 2018), CA-Net (Gu et al., 2020), TransFuse (Zhang et al., 2021) and TransUNet (Chen et al., 2021). On the ISIC-2018 dataset, U-Net (Ronneberger et al., 2015), DeepLabv3 (Chen et al., 2017), CE-Net (Gu et al., 2019), UCTransNet (Wang et al., 2022) and BAT (Wang et al., 2021) are compared with our model respectively. All models are experimented in the same environment as our proposed model.

As shown in Table 2, it can be found that our model achieves the best segmentation performance for the ISIC-2016&PH2 test set. In particular, compared with the second best TransUNet, our model increases DIC and JAC by 1% and 1.8%, which proves that our model has excellent segmentation performance. Moreover, considering that the PH2 dataset is used as a test set and has not been introduced into the training of the model, SL-HarDNet still achieves excellent performance, indicating that our model has strong generalization ability.

**TABLE 2:** Skin lesion segmentation performance of our SL-HarDNet and several popular segmentation methods on the ISIC-2016&PH2 test set and ISIC2018 dataset.

Datasets	Methods	DIC	JAC	ACC	SEN	SPE
ISIC-2016&PH2	FCN	0.889	0.811	0.932	0.967	0.922
	U-Net++	0.910	0.844	0.937	0.925	**0.960**
	CA-Net	0.894	0.819	0.936	0.938	0.947
	TransFuse	0.914	0.850	0.945	0.972	0.919
	TransUNet	0.917	0.853	0.942	0.968	0.915
	SL-HarDNet (Ours)	**0.927**	**0.871**	**0.953**	**0.975**	0.926
ISIC-2018	U-Net	0.848	0.769	0.945	0.881	0.964
	DeepLabv3	0.894	0.825	0.962	0.910	0.967
	CE-Net	0.906	0.839	0.969	0.916	0.976
	UCTransNet	0.910	0.849	0.971	0.920	0.976
	BAT	0.911	0.848	0.971	0.925	0.974
	SL-HarDNet (Ours)	**0.915**	**0.853**	**0.972**	**0.926**	**0.980**

The bold value is to emphasize that this value is optimal.

At the same time, our model is further extensively evaluated through ISIC-2018 dataset. We perform 5-fold cross-validation on the ISIC-2018 dataset, and Table 2 shows the test results at 1-fold. In Table 2, our model achieves optimal values on all evaluation indexes, indicating that our model maintains stable segmentation performance under different datasets, and has excellent robustness. The segmentation results are closer to Ground Truth.

#### 4.4.2 Visualized comparison

**FIGURE 6:**
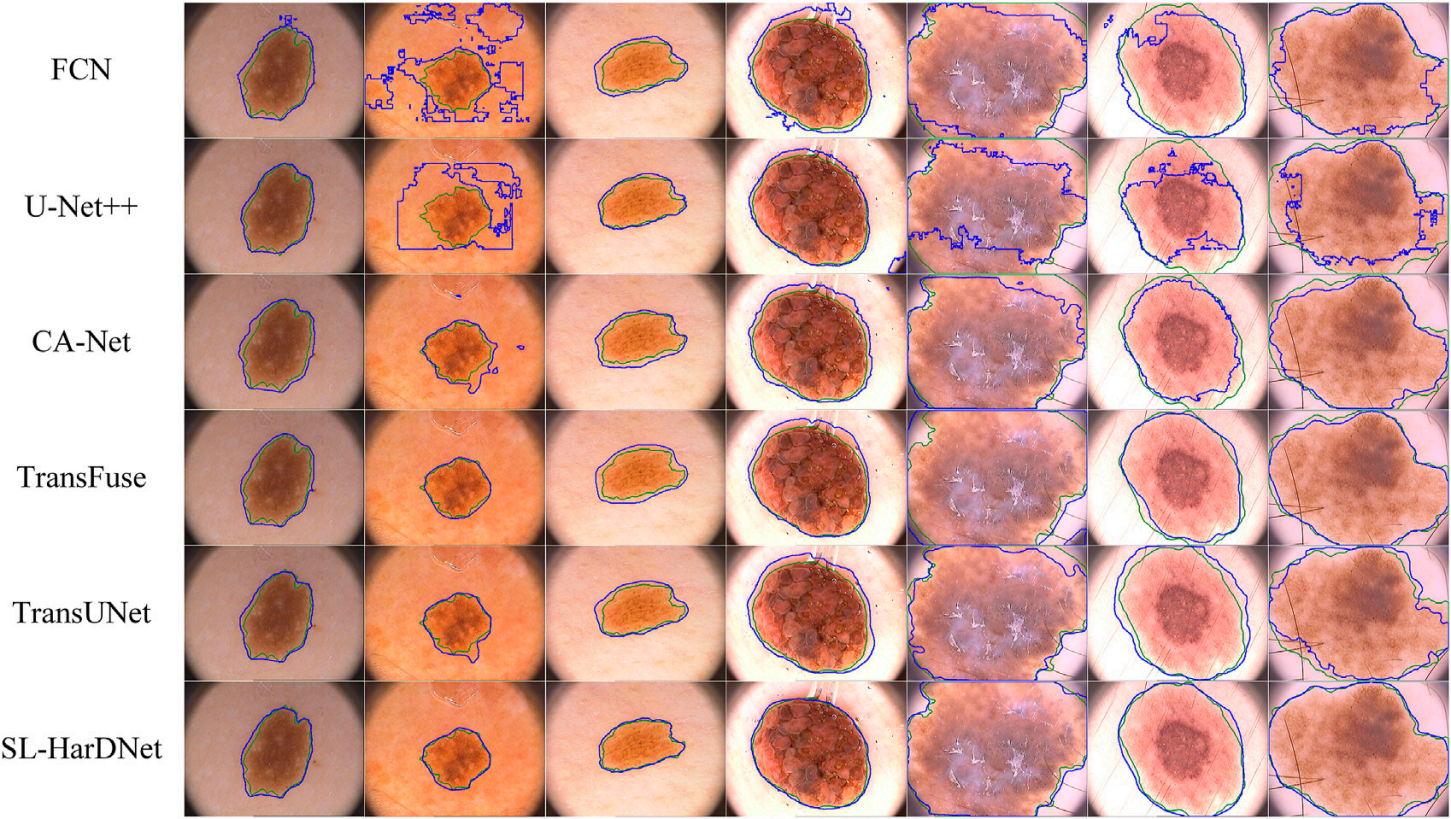
Segmentation results with contours on the ISIC-2016&PH2 test set. Ground truth and our segmentation results are shown by green and blue contours.

**FIGURE 7:**
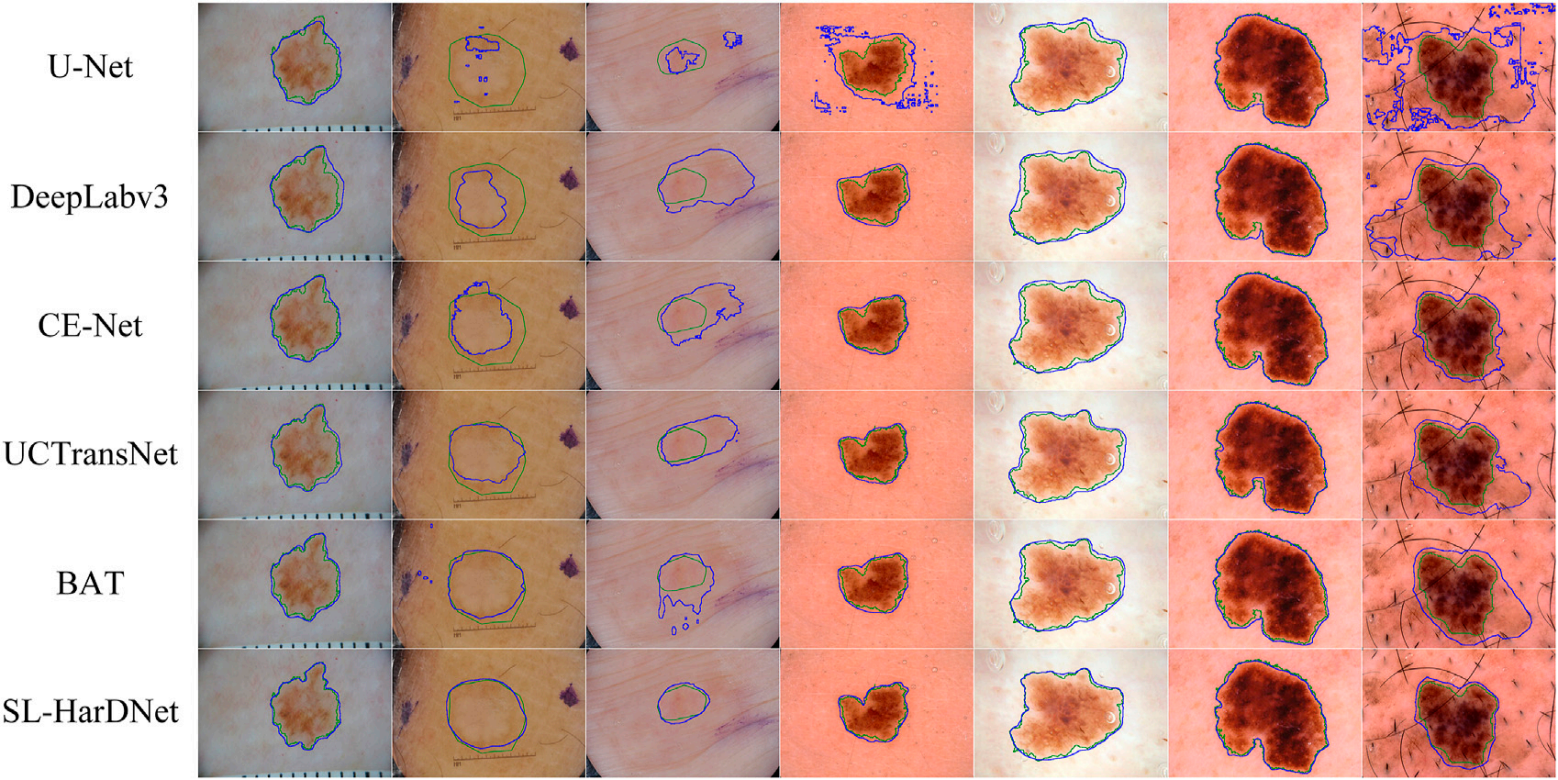
Segmentation results with contours on the ISIC-2018 dataset. Ground truth and our segmentation results are shown by green and blue contours.

From the ISIC-2016&PH2 test set and the ISIC-2018 1-fold validation dataset, we specially select some dermoscopy lesions with different sizes, irregular shapes, hair occlusion and blurred lesion boundaries, and predict and visualize these representative images, as shown in Figure 6 and Figure 7. The first three columns in Figure 6 and the first four columns in Figure 7 are small-scale lesions, and the last four columns in Figure 6 and the last three in Figure 7 are large-scale lesions. It can be found that SL-HarDNet has a stable and best prediction for lesions with different sizes and shapes. In Figure 7, the second and third columns of skin lesions have low contrast to the surrounding skin, and SL-HarDNet obtains the best boundary segmentation. For the last two columns in Figure 6 and the last column in Figure 7 with hair occlusion, our model is still optimal for boundary segmentation. The results show that our model can effectively solve the difficulty of skin lesion segmentation and obtain the optimal segmentation results.

#### 4.4.3 Ablation study

We conduct extensive ablation experiments on the ISIC-2016&PH2 test set, and describe in detail the effectiveness of each component in the overall model. Training, testing, and hyperparameter settings are consistent with those mentioned in 4.2. The results are shown in Table 3.

**TABLE 3:** Quantitative results for ablation studies on the ISIC-2016&PH2 test set.

CFM	SCAM	FAM	DIC	JAC	ACC	SEN	SPE
			0.910	0.842	0.942	0.979	0.919
**✓**			0.916	0.851	0.949	**0.981**	0.924
**✓**	**✓**		0.919	0.855	0.948	0.978	**0.926**
**✓**	**✓**	**✓**	**0.927**	**0.871**	**0.953**	0.975	**0.926**

The bold value is to emphasize that this value is optimal.

**FIGURE 8:**
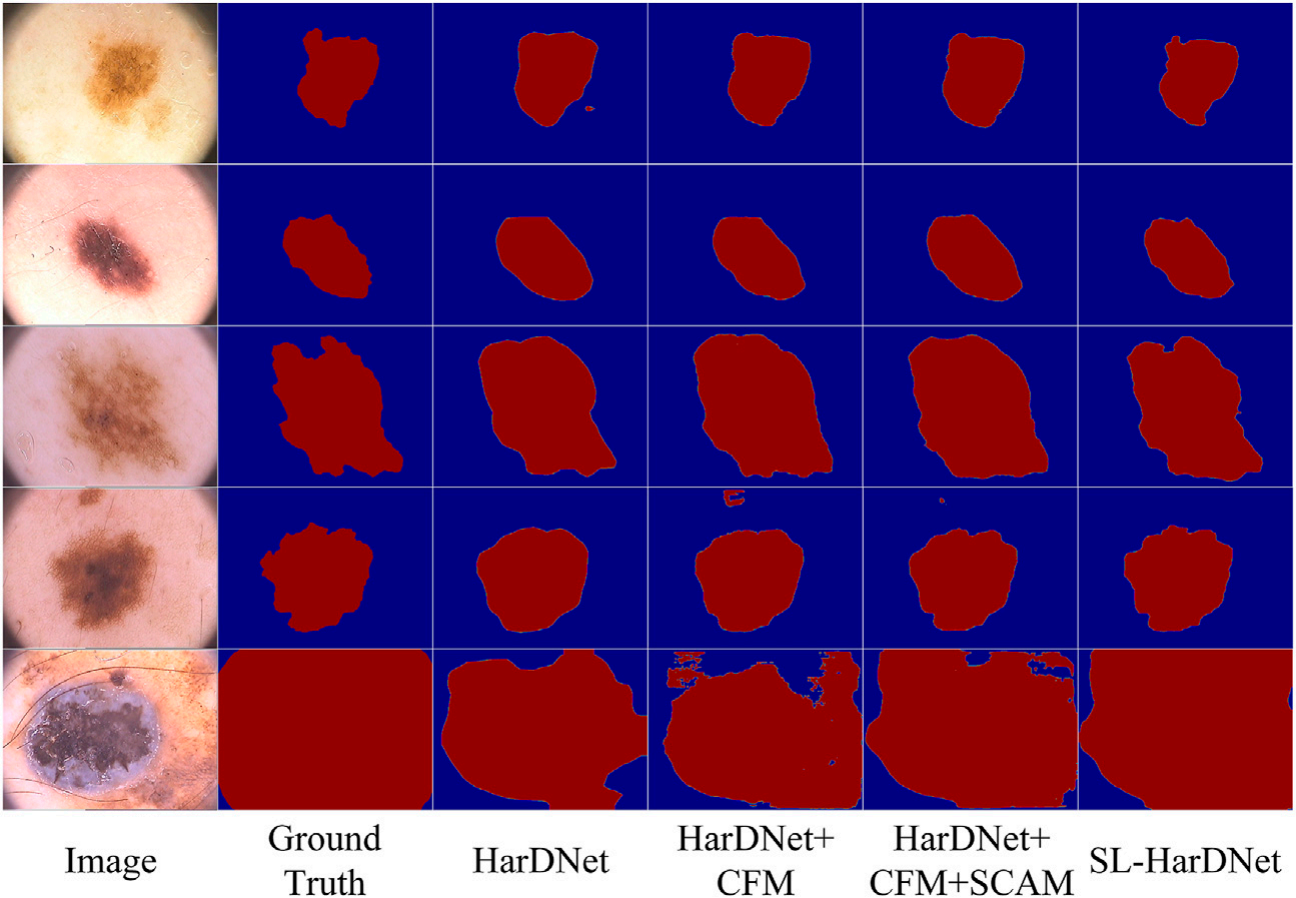
Visualization of the ablation study results.

We adopt HarDNet as baseline and remove components from the complete SL-HarDNet. At the same time, the variant is compared with the standard version to evaluate the effectiveness of the module. The standard version is denoted as “SL-HarDNet (HarDNet + CFM + SCAM + FAM)”, where “CFM”, “SCAM”, and “FAM” represent the use of components CFM, SCAM, and FAM, respectively. When baseline is selected, the values of DIC and JAC are only 91% and 84.2%. After introducing CFM into the baseline, DIC, JAC and ACC are significantly rose, especially JAC is increased by 0.9%. Then, after the introduction of SCAM, DIC is increased to 91.9% and JAC is rose to 85.5%, which are further improved. After adding FAM, other evaluation indexes except SEN are reached the optimal value, especially compared with the baseline, JAC has a 2.9% improvement. It can be found that with HarDNet as the baseline, FCM, SCAM, and FAM components are added respectively, which shows obvious performance improvement on the test set and verifies the effectiveness of each component. Then, we visualize the results of ablation studies, as shown in Figure 8. It can be observed that baseline HarDNet lacks sufficient ability to obtain fine boundaries. With the introduction of corresponding components, false detection is effectively avoided, more attention is paid to the lesion area, and the boundary is refined. Finally, the SL-HarDNet (HarDNet + CFM + SCAM + FAM) achieves the best segmentation performance.

### 4.5 Combine loss function

**FIGURE 9:**
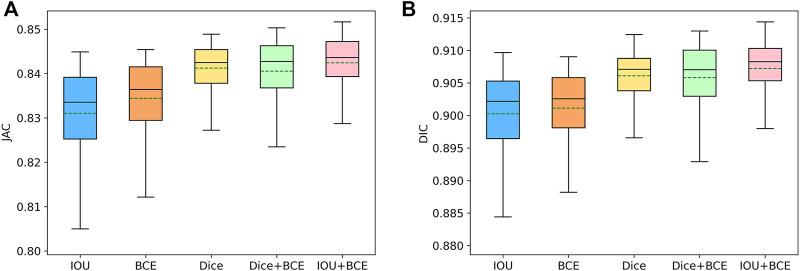
Box plots of the typical metrics. i.e., JAC **(A)** and DIC **(B)**, for different loss function in SL-HarDNet. The mean value of each metric is represented by a green dashed line.

**FIGURE 10:**
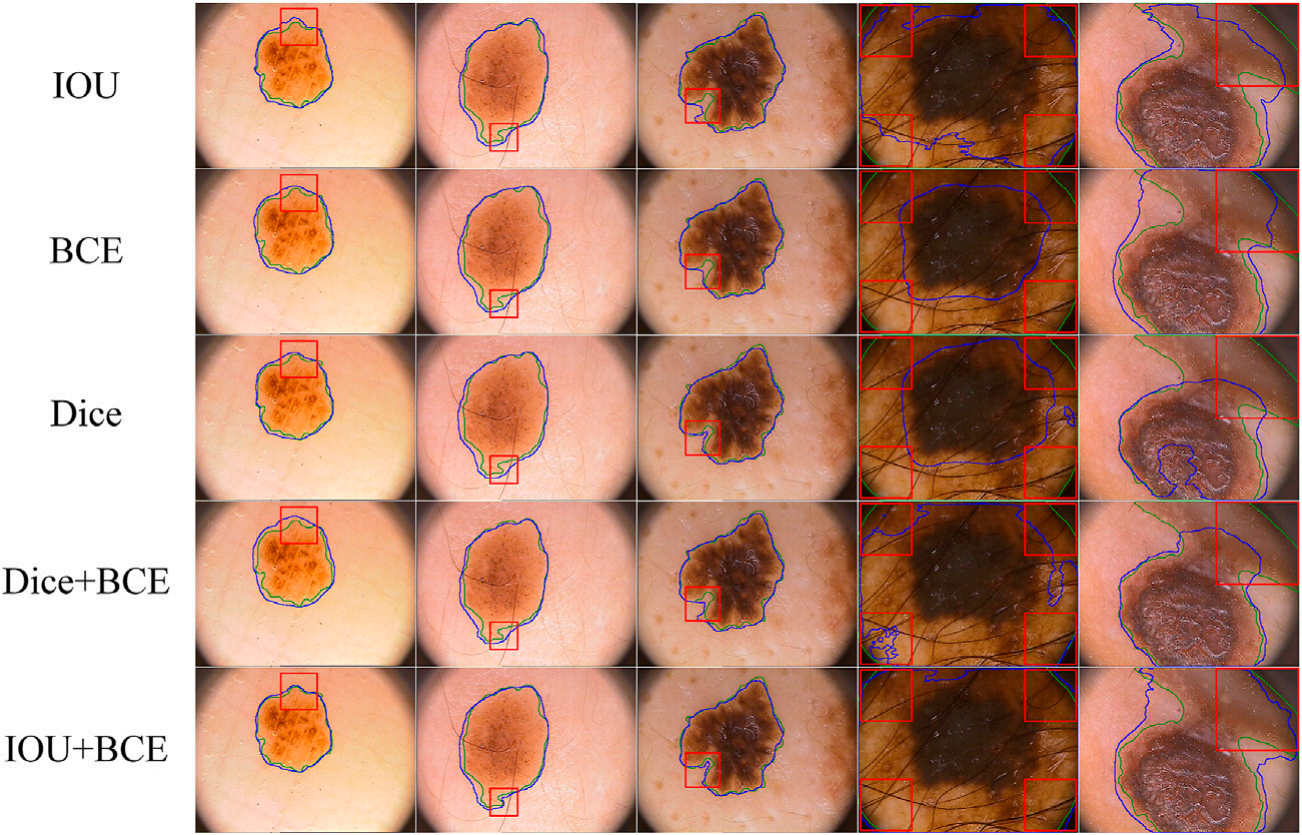
Visualize segmentation results obtained by different loss functions. Uncertain and challenging boundaries are marked through red boxes.

On the ISIC-2018 dataset, we evaluate the contribution of the combined loss functions and compare them with IOU, BCE and Dice loss functions. As shown in Table 4, IOU is closest to BCE on DIC and JAC values. Dice is superior to IOU and BCE, which significantly improves the segmentation performance. Using the combination of Dice and BCE loss functions, the results of DIC and JAC are further improved, but the SPE values of Dice and BCE are reduced. When a combination of IOU and BCE is used, the values of DIC, JAC and ACC are further increased, significantly better than other functions. In addition, we use box plots to analyze the performance of each loss function. As shown in Figure 9, we find that the first quartile ( $Q_{1}$ ), median ( $Q_{2}$ ), last quartile ( $Q_{3}$ ), minimum, maximum and mean values of Dice are larger than BCE and Dice. The combination of Dice and BCE has been improved in $Q_{2}$ , $Q_{3}$ and maximum, but $Q_{1}$ , minimum and mean values are significantly less than Dice. For the combination of IOU and BCE, all values are improved, significantly better than other loss functions, which proves that the combination function is effective. From the above comparison, the combination of IOU and BCE achieves better segmentation performance when dealing with class imbalance problems. Furthermore, in order to verify the effectiveness of IOU and BCE combination loss, we visualize it on the ISIC-2016&PH2 test set. As shown in Figure 10, the combination loss of IOU and BCE obtains the best segmentation result. Especially for the region with uncertain boundary, the boundary segmentation is clearer and more complete, and the segmentation result is closer to Ground Truth.

**TABLE 4:** Different loss functions in SL-HarDNet using the ISIC-2018 skin lesion segmentation.

Methods	DIC	JAC	ACC	SEN	SPE
IOU	0.909	0.844	0.970	**0.932**	0.979
BCE	0.908	0.845	0.971	0.910	**0.980**
Dice	0.912	0.849	0.971	0.919	0.977
Dice + BCE	0.913	0.850	0.970	0.921	0.975
IOU + BCE	**0.915**	**0.853**	**0.972**	0.926	**0.980**

The bold value is to emphasize that this value is optimal.

## 5 Conclusion

In this paper, we propose a novel skin lesion segmentation model termed SL-HarDNet, which adopts HarDNet as the backbone network and can extract strong semantic features. At the same time, we introduce three components with excellent performance, namely Cascaded Fusion Module (CFM), Spatial Channel Attention Module (SCAM) and Feature Aggregation Module (FAM), which effectively collect high-level semantic and low-level spatial information, and mine local and global semantic clues, and finally fuse them to obtain output. We conduct comparative experiments on the datasets of two skin lesions to effectively verify the segmentation accuracy and generalization ability of SL-HarDNet. The results show that our model is consistently superior to all contrasting models. Although our model is based on specific applications of skin lesion segmentation, in future work, we can apply our components to other medical image segmentation tasks based on deep learning to improve segmentation performance.

## Data Availability

Publicly available datasets. ISIC-2016 and ISIC-2018 can be found through https://challenge.isic-archive.com/data/, PH2 can be found through https://www.fc.up.pt/addi/ph2%20database.html.
